# PIAS1 interacts with FLASH and enhances its co-activation of c-Myb

**DOI:** 10.1186/1476-4598-10-21

**Published:** 2011-02-21

**Authors:** Anne Hege Alm-Kristiansen, Petra I Lorenzo, Ann-Kristin Molværsmyr, Vilborg Matre, Marit Ledsaak, Thomas Sæther, Odd S Gabrielsen

**Affiliations:** 1 Department of Molecular Biosciences, University of Oslo, N-0316 Oslo, Norway; 2 Current Address: BioKapital AS, N-2317 Hamar, Norway; 3 Current Address: Department of Stem Cells, Andalusian Center for Molecular Biology and Regenerative Medicine, Seville, Spain

## Abstract

**Background:**

FLASH is a huge nuclear protein involved in various cellular functions such as apoptosis signalling, NF-κB activation, S-phase regulation, processing of histone pre-mRNAs, and co-regulation of transcription. Recently, we identified FLASH as a co-activator of the transcription factor c-Myb and found FLASH to be tightly associated with active transcription foci. As a huge multifunctional protein, FLASH is expected to have many interaction partners, some which may shed light on its function as a transcriptional regulator.

**Results:**

To find additional FLASH-associated proteins, we performed a yeast two-hybrid (Y2H) screening with FLASH as bait and identified the SUMO E3 ligase PIAS1 as an interaction partner. The association appears to involve two distinct interaction surfaces in FLASH. We verified the interaction by Y2H-mating, GST pulldowns, co-IP and ChIP. FLASH and PIAS1 were found to co-localize in nuclear speckles. Functional assays revealed that PIAS1 enhances the intrinsic transcriptional activity of FLASH in a RING finger-dependent manner. Furthermore, PIAS1 also augments the specific activity of c-Myb, and cooperates with FLASH to further co-activate c-Myb. The three proteins, FLASH, PIAS1, and c-Myb, are all co-localized with active RNA polymerase II foci, resembling transcription factories.

**Conclusions:**

We conclude that PIAS1 is a common partner for two cancer-related nuclear factors, c-Myb and FLASH. Our results point to a functional cooperation between FLASH and PIAS1 in the enhancement of c-Myb activity in active nuclear foci.

## Background

The FLICE associated huge protein (FLASH) has been reported to be a potential prognostic marker in cases of acute lymphoblastic leukaemia [1] and recently also detected as a novel partner gene of MLL rearrangement in acute myeloid leukaemia [2]. FLASH was originally identified as a caspase-8 interacting protein and was reported to be necessary for the activation of caspase-8 in Fas-mediated apoptosis [3]. More recent findings suggest that FLASH may have a role in apoptosis by being part of a nuclear signalling pathway involving the PML nuclear body component Sp100 [4]. Consistent with a nuclear function, FLASH was found localized mainly in nuclear speckles, partially co-localizing with Cajal bodies and PML nuclear bodies [4-6]. Nevertheless, FLASH may still have a temporary cytoplasmic function as it appears to shuttle from the nucleus to the cytoplasm in a caspase-dependent process upon CD95 activation [4]. Interestingly, functional studies have shown that apart from its role as a pro-apoptotic protein, FLASH is also involved in control of cell cycle progression. Down-regulation of FLASH reduced histone gene transcription and caused a block of cells within S-phase of the cell cycle [7]. This function of FLASH was recently appointed to its association with Histone Locus Bodies (HLBs) [8]. FLASH is essential for 3' processing of histone pre-mRNAs taking place in HLBs [9], and a disruption of these bodies leads to a cell-cycle arrest [10]. Importantly, the role of FLASH in transcriptional control is not limited to histones. FLASH appears to have a cell type specific function as a regulator of steroid hormone receptor signalling. FLASH causes down-regulation of GR, PR and AR in a colon carcinoma cell line [11,12], but activates GR and MR in hippocampal neurons [13]. We have earlier demonstrated a new function of FLASH as a co-activator of the transcription factor c-Myb [6]. We showed that FLASH enhanced the expression of the endogenous c-Myb target gene *mim-1 *and knock-down of FLASH resulted in a reduction in expression of c-Myb target genes in haematopoietic cells [6]. All these diverse roles point to FLASH as being a multifunctional nuclear protein.

The transcription factor c-Myb plays a central role in the regulation of cell growth and differentiation in haematopoietic cells [14]. It operates as a regulator of stem and progenitor cells in the bone marrow, as well as in colonic crypts and in a neurogenic region of the adult brain [15]. In the avian system, leukaemia is induced by truncated and mutated forms of v-Myb encoded by two types of retrovirus strains (AMV and E26). The link between *MYB *aberrations and human cancer was recently strengthened by the detection of duplications and translocations of the *MYB *gene in T cell acute lymphoblastic leukaemia [16,17]. Moreover, Stenman and co-workers reported that a *MYB*-*NFIB *fusion is a hallmark of adenoid cystic carcinomas (ACC) of the breast, head and neck, and that deregulation of the expression of *MYB *is a key oncogenic event in the pathogenesis of ACC [18]. The emerging picture is that the level of c-Myb seems to be critical for proper functioning of the haematopoietic system and in other tissues where c-Myb plays a key role, and only a modest deregulation may have dramatic biological effects. The activity of c-Myb is controlled by the expression levels of the protein, through mechanisms where miRNAs seem to play a major role [18,19]. In addition, c-Myb is also modulated by post-translational modifications as well as by protein-protein interactions. c-Myb is phosphorylated at several sites by different kinases [14,20-23], sumoylated at two lysine residues in the C-terminal regulatory domain [24-26] and acetylated by the co-activator CBP/p300 [27,28]. Moreover, different factors have been reported to interact with c-Myb regulating its activity; besides FLASH [6], we have also earlier identified another co-activator of c-Myb, the chromatin remodelling factor Mi-2α, shown to regulate endogenous c-Myb target genes [29]. Recently, we also showed that interaction with SUMO is involved in regulation of c-Myb activity [30].

The protein inhibitor of activated STAT (PIAS) family of proteins consists of PIAS1, PIAS2 (PIASx), PIAS3 and PIAS4 (PIASy) that share a high degree of sequence conservation (reviewed in [31,32]), and hZimp7 and hZimp10 with more limited sequence similarity [33,34]. In addition to their function as negative regulators of STAT signalling, PIAS proteins can also act as SUMO E3 ligases, enhancing sumoylation of target proteins [31,32]. This activity is dependent on a RING finger domain present in PIAS proteins [31,32,35]. Since sumoylation of transcriptional regulators often leads to inhibition of their activity [36,37], PIAS proteins have been described as negative regulators of transcription. However, PIAS proteins are also known to act as co-activators [31]. It has also been reported that PIAS proteins can bind to and alter the subcellular localization of different proteins [38-40]. Interestingly, these activities of PIAS proteins as modulators of transcription may occur both in SUMO-dependent or independent manners [32].

In an effort to better understand FLASH action, we searched for new interaction partners for FLASH. In a yeast two-hybrid screening using FLASH as bait, we identified PIAS1 as a binding partner. Here we show that PIAS1 enhances FLASH activity and its ability to co-activate c-Myb. PIAS1 together with FLASH is able to further enhance the transcriptional activity of c-Myb. Consistent with the up-regulation of activity of both FLASH and c-Myb, all three proteins, FLASH, c-Myb and PIAS1, are co-localized in active RNA polymerase II foci. These results suggest that FLASH and PIAS1 cooperate in enhancing c-Myb transcriptional activity in foci that resemble transcription factories.

## Results

### FLASH interacts with PIAS1

**Figure 1:**
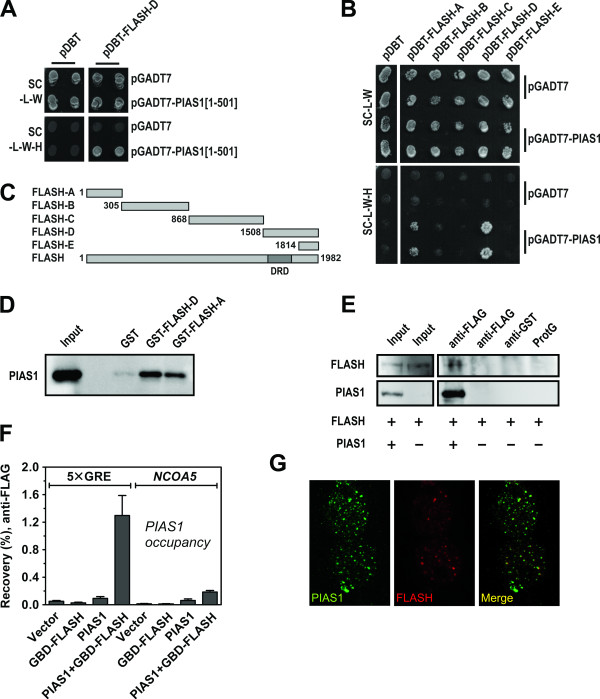
**FLASH and PIAS1 interact and co-localize in nuclear speckles**. **(A) **AH109 yeast cells were transformed with bait vector pDBT or pDBT-FLASH-D and mated with the yeast strain Y187 pretransformed with prey vector pGADT7 or pGADT7-PIAS1[1-501]. Interaction was verified by activation of the *HIS3 *reporter gene (SC-L-W-H). Each mating was performed in duplicate. **(B) **AH109 yeast cells were transformed with bait vector pDBT or pDBT-FLASH-A/B/C/D/E and mated with Y187 pretransformed with prey vector pGADT7 or pGADT7-PIAS1. Interaction was verified by activation of the *HIS3 *reporter gene (SC-L-W-H). Each mating was performed in duplicate. **(C) **Schematic overview of the regions of FLASH used in yeast two-hybrid assays. Abbreviation; DRD = DED-recruiting domain [3]. A: amino acids 1-304, B: 305-867, C: 868-1507, D: 1508-1982, E: 1814-1982. **(D) **GST, GST-FLASH-D and GST-FLASH-A were incubated with lysate from COS-1 cells transfected with full-length 3×FLAG-tagged PIAS1. PIAS1 was detected by an anti-FLAG antibody. 5% of the input (total cell extract) used for the pulldown was loaded as reference. **(E) **Lysates from COS-1 cells transfected with GFP-FLASH and 3×FLAG-PIAS1 or empty vector, were immunoprecipitated with anti-FLAG antibody or an irrelevant antibody (anti-GST), and the precipitates were analyzed by immunoblotting using anti-GFP (FLASH) and anti-FLAG (PIAS1) for detection. 5% (PIAS1) or 10% (FLASH) of the input used for immunoprecipitation was loaded as reference. ProtG, protein G-Sepharose beads only. **(F) **HEK 293 cells were transfected with plasmids expressing 3×FLAG-PIAS1 and a Gal4p-DBD-FLASH fusion protein. Occupancies of PIAS1 on the 5×GRE promoter and on the *NCOA5 *intron were analysed using ChIP-qPCR. **(G) **CV-1 cells were transfected with plasmids encoding HA-PIAS1 and 3×FLAG-FLASH, and analysed with indirect immunofluorescence and confocal microscopy. PIAS1 was detected with rabbit anti-HA antibody and Alexa Fluor 488 goat anti-rabbit IgG. FLASH was detected with mouse anti-FLAG antibody and Alexa Fluor 633 goat anti-mouse IgG1. The merged image is shown in the right panel.

To advance our understanding of FLASH function, we performed a yeast two-hybrid (Y2H) screening with FLASH as bait, using a human bone marrow cDNA library (Clontech). Due to the high level of autoactivation of full-length FLASH, even when using the centromeric low copy vector pDBT, we selected as bait a C-terminal fragment of FLASH encoding amino acid residues 1508-1982 (FLASH-D). This part of the protein contains the DED-recruiting domain (DRD) [3] and also includes the region that interacts with c-Myb [6]. The N-terminal border of this fragment was placed in a serine/proline-rich area, predicted to be located between globular domains http://globplot.embl.de. Among the positive clones obtained in the Y2H screening we identified the SUMO E3 ligase PIAS1 (amino acids 1-501). The interaction between FLASH-D and PIAS1 was verified by retransformation in yeast and testing for activation of the *HIS3 *(Figure 1A) and *LacZ *(not shown) reporter genes. Further Y2H mating assays using FLASH-ΔD (amino acid residues 1- 1507) as bait indicated that PIAS1[1-501] did not interact with other regions of FLASH (data not shown). On the other hand, full length PIAS1 was able to interact not only with FLASH-D, but also with the N-terminal fragment of FLASH (amino acid residues 1-304), FLASH-A (Figure 1B and 1C). This indicates that the C-terminal part of PIAS1 probably represents a second interaction surface, associating with the N-terminal part of FLASH.

The interaction between FLASH and PIAS1 was confirmed by GST pulldown assays. Specific binding was observed when GST-FLASH-D and GST-FLASH-A was incubated with lysates from COS-1 cells transfected with full-length PIAS1 (Figure 1D). These results support the data obtained from the Y2H assays, confirming the interaction between PIAS1 and both N- and C-terminal regions of FLASH. A third line of evidence for the interaction was provided by co-immunoprecipitation assays using lysates from COS-1 cells transfected with full-length FLASH and PIAS1. As shown in Figure 1E, FLASH was co-immunoprecipitated with FLAG-tagged PIAS1 using anti-FLAG antibodies, but not with anti-GST antibodies or Sepharose beads. Finally, we took advantage of a reporter cell line with an integrated Gal4p-responsive promoter to study this interaction in a chromatin context [41]. Using chromatin immunoprecipitation (ChIP) we did not see any enrichment of PIAS1 on the promoter when expressed alone (Figure 1F). However, when transfected together with FLASH fused to a Gal4p DNA-binding domain, PIAS1 was efficiently recruited to the GAL promoter through Gal-FLASH. This was not seen for the neighbouring *NCO5A *control promoter (Figure 1F). Altogether this shows that PIAS1 and FLASH interact, also in a chromatin context.

### FLASH and PIAS1 co-localize in nuclear speckles

Both FLASH and PIAS1 have been found localized mainly in nuclear speckles in several cell lines [4-6,39,42,43]. If an interaction between FLASH and PIAS1 exists, we would expect, at least, a partial co-localization of the speckles in which these proteins are found. To examine this, we transfected CV-1 cells with HA-tagged FLASH and FLAG-tagged PIAS1 and analyzed their subcellular localization by immunofluorescence and confocal microscopy. Consistent with previous reports [4-6,42-44], we found that both PIAS1 and FLASH were localized in nuclear foci (Figure 1G). Whereas PIAS1 was distributed both in the nucleoplasm and in nuclear speckles (Figure 1G, left panel) and was found in a larger number of speckles than FLASH, it is evident that the majority of the FLASH foci co-localized with PIAS1 foci. These observations clearly support the notion that FLASH and PIAS1 proteins are able to co-localize in mammalian cells, consistent with their mutual binding affinities.

### The function of PIAS1 in relation to FLASH

In order to determine the functional consequences of the PIAS1-FLASH interaction, we addressed two main questions: 1) Whether PIAS1 enhances FLASH sumoylation and 2) whether PIAS1 modulates the intrinsic transactivation function of FLASH.

**Figure 2:**
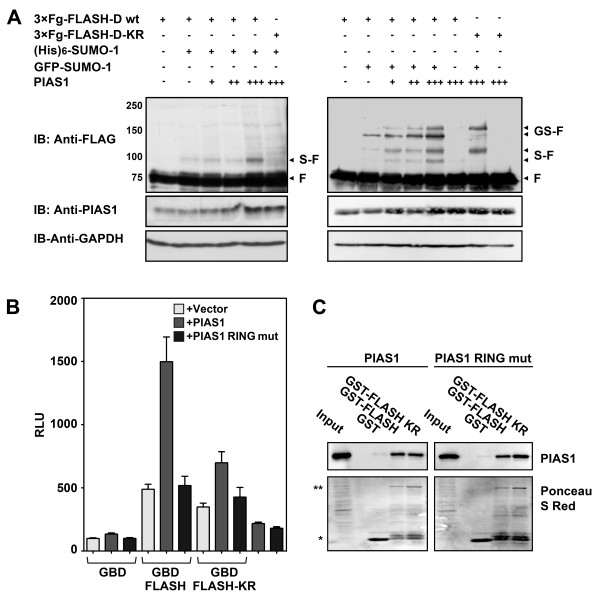
**PIAS1 functions as a SUMO E3 ligase and co-activator of FLASH**. **(A) **CV-1 cells were transfected with plasmids encoding 3×FLAG-FLASH-D or 3×FLAG-FLASH-D-KR (0.5 μg), (His)_6_-SUMO-1 (0.5 μg) and increasing amounts (0.5, 1.0, 1.5 μg) of HA-PIAS1 (left panel). Likewise, CV-1 cells were transfected with the same plasmids, except for (His)_6_-SUMO-1 which was exchanged with 0.5 μg GFP-SUMO-1 (right panel). Whole cell extracts were analyzed by SDS-PAGE and immunoblotting using anti-FLAG antibody for the detection of FLASH, as well as anti-PIAS1 and anti-GAPDH antibodies. Arrows indicate non-sumoylated FLASH (F), sumoylated FLASH (S-F), and FLASH sumoylated with GFP-SUMO1 (GS-F). **(B) **CV-1 cells were transfected with plasmids encoding Gal4p-DBD-FLASH wild-type, K1813R mutant (FLASH-KR), or Gal4p-DBD only (0.4 μg) in absence and presence of PIAS1 wild-type or PIAS1 RING finger mutant (PIAS1-C350S) (0.2 μg) in combination with a Gal4p-driven SNRPN promoter reporter construct. The results are presented as relative luciferase units (RLU) and represent the mean RLU ± SEM of three independent assays performed in triplicates. **(C) **GST, GST-FLASH-D wild-type (GST-FLASH) and K1813R (GST-FLASH KR) were incubated with lysates from COS-1 cells transfected with full-length 3×FLAG-tagged PIAS1 wild-type or RING finger mutant (C346S/C351S/H353A/C356S). PIAS1 was detected by an anti-FLAG antibody. 5% of the input (total cell extract) used for the pulldown was loaded as reference. The amount of GST and GST fusion proteins was evaluated with Ponceau S red staining of the membrane after immunoblotting. */**, GST or GST-FLASH-D, respectively.

PIAS1 is a SUMO E3 ligase, and since it interacts with FLASH, we first analyzed whether FLASH sumoylation is enhanced as a result of this interaction. We have previously shown that FLASH interacts with Ubc9 and becomes sumoylated on lysine 1813 [45]. We therefore examined whether PIAS1 stimulates SUMO-conjugation on this lysine. As shown in Figure 2A (left panel), a slower migrating band at about 100 kD appeared when co-transfecting FLASH-D with PIAS1 and SUMO-1. This band disappeared with the K1813R mutant, as expected for FLASH being sumoylated on this lysine. The effects observed when co-transfecting with GFP-SUMO-1 (Figure 2A, right panel) corroborated this interpretation. GFP-SUMO-1 both shifts the equilibrium towards sumoylated species and as a result induces new GFP-SUMO-1-FLASH bands (GS-F) migrating more slowly than the SUMO-induced shift (S-F). As can be seen in the right panel, two of the bands corresponding to SUMO-1- (S-F) and GFP-SUMO-1-modified FLASH (GS-F) disappeared with the K1813R mutant (Figure 2A). This supports the notion that FLASH is modified by SUMO on K1813. The remaining bands indicate that FLASH is sumoylated on at least one additional lysine residue as previously reported [45]. Taken together PIAS1 seems to function as a SUMO E3 ligase enhancing the sumoylation of FLASH.

Since PIAS proteins appear to operate as transcriptional co-regulators, being either activating or repressive (reviewed in [31,32]), we investigated whether PIAS1 would modulate the intrinsic transactivation function of FLASH. We performed a Gal4-tethering assay and measured the activity of Gal4p-DBD-FLASH in the absence and presence of co-transfected PIAS1. Interestingly, PIAS1 enhanced the transactivation function of FLASH about threefold in this assay (Figure 2B). No alteration of the control Gal4p-DBD activity was observed, confirming the specificity of PIAS1 action on FLASH activity. To examine whether the PIAS1 SUMO E3 ligase activity was required for the response, we performed the same type of experiment using a PIAS1 RING finger mutant that is unable to stimulate sumoylation. The RING finger mutant did not enhance the transcriptional activity of FLASH (Figure 2B). Notably, this observation suggests that PIAS1 E3 ligase activity is required for enhancing the intrinsic activity of FLASH. To address whether the presumed PIAS1 sumoylation target was FLASH, we included a Gal4p-DBD-FLASH fusion protein in which the major sumoylation site was mutated (K1813R) [45]. PIAS1 still activated FLASH-KR but to a lesser extent than FLASH wild-type (Figure 2B). As expected, the PIAS1 RING finger mutant did not enhance the FLASH-KR activity (Figure 2B). None of these effects were due to altered interactions. As seen in Figure 2C, PIAS1 with the RING finger mutated bound FLASH with the same efficiency as PIAS1 wild-type. Similarly, the K1813R mutation in the SUMO acceptor lysine of FLASH had no effect on the interaction with PIAS1, wild-type or RING finger mutant (Figure 2C). Taken together, these data imply that PIAS1 acts as a co-activator of FLASH in a RING finger-dependent manner, and that sumoylation of FLASH is required for full enhancement of FLASH activity.

### Regulation of c-Myb activity by PIAS1 and FLASH

**Figure 3:**
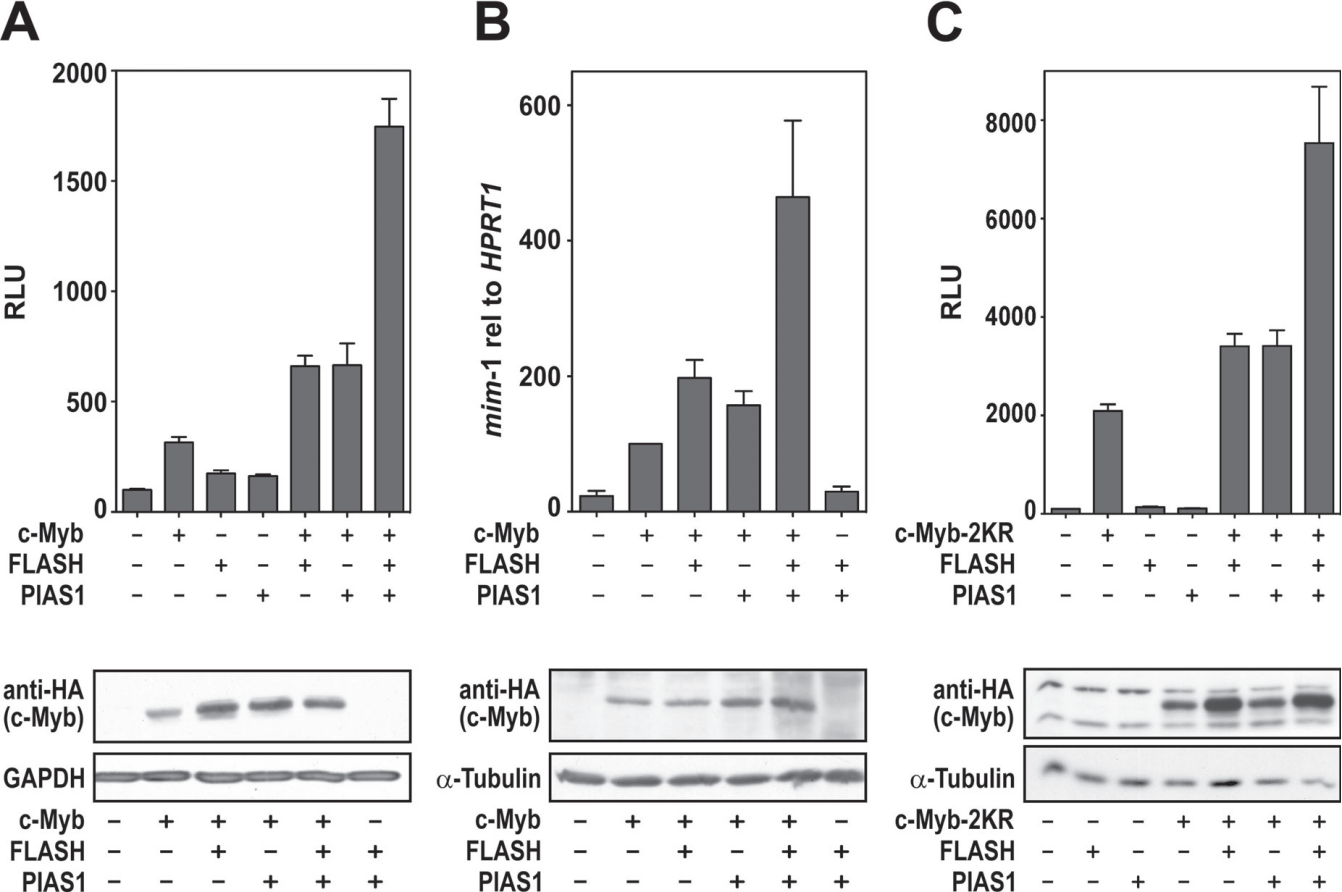
**Regulation of c-Myb activity by PIAS1 and FLASH**. **(A) **CV-1 cells were transfected with the c-Myb-responsive 3×MRE(GG)-MYC reporter plasmid (0.2 μg) and plasmids encoding c-Myb, FLASH and PIAS1 as indicated (each 0.2 μg). **(B) **HD11 cells were transfected with plasmids encoding c-Myb, FLASH and PIAS1 as indicated. c-Myb activation of the target gene *mim-1 *was measured by quantitative real-time PCR with primers specific for *mim-1 *and *HPRT*. The results are presented as *mim-1 *expression relative to HPRT expressions ± SEM. **(C) **CV-1 cells were transfected with the c-Myb-responsive 3×MRE(GG)-MYC reporter plasmid (0.2 μg) and plasmids encoding c-Myb-2KR, FLASH and PIAS1 as indicated (0.2 μg each). The results in (A) and (C) are presented as relative luciferase units (RLU) and represent the mean RLU ± SEM of three independent assays performed in triplicates. For each experiment Western immunoblotting is shown below.

Our previous studies had shown that FLASH binds to c-Myb and enhances c-Myb dependent target gene activation [6]. Having found that PIAS1 enhances the activity of FLASH (Figure 2), we next asked whether the interaction of PIAS1 with FLASH had any influence on the activity of c-Myb. Reporter assays showed that PIAS1 enhanced c-Myb activity to about the same degree as FLASH (Figure 3A). Moreover, PIAS1 and FLASH together enhanced c-Myb dependent transcription even further, implying that they cooperate to increase c-Myb dependent activity (Figure 3A). In order to determine if these results could be extended to a more physiological system, we also analyzed the expression of an endogenous c-Myb target gene, *mim-1*, in the haematopoietic cell line HD11. As shown in Figure 3B, both FLASH and PIAS1 enhanced c-Myb-dependent expression of *mim-1*, and the co-expression of both proteins induced a further increase in the expression levels of *mim-1*, very similar to what was observed in the reporter assays (Figure 3A). These results indicate that PIAS1 and FLASH cooperate in the enhancement of c-Myb transcriptional activity.

For PIAS1-mediated co-activation of FLASH we found that PIAS1 required an intact SUMO E3 ligase activity (Figure 2). Therefore, we asked whether the PIAS1-mediated activation of c-Myb was dependent on c-Myb sumoylation. c-Myb is sumoylated in lysine 503 and 527 [24,25]. The mutation of both these lysines (c-Myb-2KR) completely abolishes c-Myb sumoylation and creates a significantly more active factor. We found that PIAS1 still activated the SUMO-negative c-Myb-2KR to about the same degree as wild-type c-Myb (Figure 3C). The observation that PIAS1 activates c-Myb independent of SUMO-status is consistent with an earlier report stating that PIAS1 does not appear to have any significant effect on the sumoylation of c-Myb [46]. Taken together, these results suggest that even though an intact PIAS1 E3 RING finger domain is required for enhancement of FLASH transactivation, PIAS1-mediated co-activation of c-Myb seems to be independent on c-Myb sumoylation.

**Figure 4:**
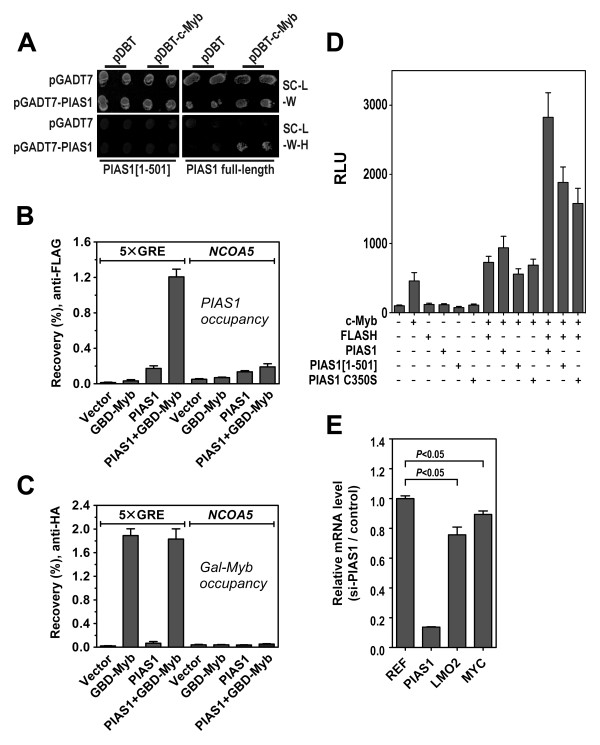
**PIAS1 interacts with c-Myb on chromatin and activates the expression of c-Myb target genes**. **(A) **AH109 yeast cells were transformed with bait vector pDBT or pDBT-c-Myb and mated with the yeast strain Y187 pretransformed with pray vector pGADT7, pGADT7-PIAS1[1-501] or pGADT7-PIAS1. Interaction was verified by activation of the *HIS3 *reporter gene (SC-L-W-H). Each mating was performed in duplicate. HEK 293 cells were transfected with plasmids expressing 3×FLAG-PIAS1 and a Gal4p-DBD-c-Myb-HA fusion protein. Occupancies of **(B) **PIAS1 and **(C) **c-Myb on the 5×GRE promoter and on the *NCOA5 *intron were analysed using ChIP-qPCR. **(D) **CV-1 cells were transfected with the c-Myb-responsive 3×MRE(GG)-MYC reporter plasmid (0.2 μg) and plasmids encoding c-Myb, FLASH, PIAS1, PIAS1[1-501] or PIAS1[C350S] as indicated (0.2 μg each). The results are presented as relative luciferase units (RLU) and represent the mean RLU ± SEM of three independent assays performed in triplicates. **(E) **K562 cells were transfected with siRNAs directed against PIAS1. Effects of PIAS1 knock-down on the endogenous c-Myb target genes *MYC *and *LMO2 *were measured by quantitative real-time PCR using specific primers for the target genes and the reference genes *ACTB *and *POLR2A*. The results are presented as *PIAS1*, *MYC *or *LMO2 *expression (normalized for *ACTB *and *POLR2A *expression) after PIAS1 knock-down relative to their expressions in the knock-down control. The results are presented as mean relative mRNA level ± SEM. Statistical significance was calculated using the Student's *t*-test (*P*-value indicated).

To further understand the role of PIAS1 in the enhancement of FLASH-mediated co-activation of c-Myb, we tested whether PIAS1 and c-Myb interact directly. A Y2H mating assay indicated that c-Myb binds full-length PIAS1. Interestingly, it does not appear to bind the shorter version PIAS1[1-501] (Figure 4A), suggesting that the C-terminal 150 amino acid residues of PIAS1 are necessary for its c-Myb interaction. We also used the above-mentioned reporter cell line and ChIP as an independent assay of this interaction on chromatin and observed that PIAS1 was recruited to the reporter promoter only in the presence of c-Myb (Figure 4B). This was not caused by PIAS1 affecting the Myb ChIP, as c-Myb co-immunoprecipitated the promoter just as efficiently when transfected alone as when co-transfected with PIAS1 (Figure 4C).

An interaction between c-Myb and PIAS1 in addition to the one between PIAS1 and FLASH may stabilize the c-Myb-FLASH interaction. Given that a triple complex is the most active form, this may explain why PIAS1, together with FLASH, further enhances the transcriptional activity of c-Myb (Figure 3). To analyze this, we studied the effect of both PIAS1 full length and the shorter version PIAS1[1-501] in a c-Myb dependent reporter assay. As previously observed, both FLASH and PIAS1 individually activated c-Myb. On the other hand, PIAS1[1-501] had only a slight effect on the activity of c-Myb (Figure 4D), consistent with lost c-Myb binding properties. However, in the presence of co-transfected FLASH, PIAS1[1-501] also became able to enhance the transcriptional activity of c-Myb, probably through the enhancement of FLASH co-activation. Nevertheless, when full length PIAS1 is co-expressed, the transactivation activity of c-Myb was further enhanced, supporting our hypothesis that the triple complex c-Myb-FLASH-PIAS1 could represent the full complex needed for maximal activity. Notably, the PIAS1 RING finger mutant, that did not enhance FLASH intrinsic activity (Figure 2B), resembled PIAS1[1-501] in its fairly small enhancement of c-Myb transcriptional activity when co-expressed with FLASH (Figure 4D).

Finally, we reasoned that if PIAS1 acts as one of the co-activators of c-Myb, one would expect to see an effect on endogenous target genes of c-Myb if the level of PIAS1 was significantly reduced. To address this, we specifically knocked down PIAS1 in the c-Myb expressing human erythroleukaemia K562 cells and monitored the expression of two established c-Myb target genes, *MYC *and *LMO2 *[47,48]. As the mRNA of PIAS1 dropped to only ~14% of its normal level, the two target genes *MYC *and *LMO2 *were both significantly down-regulated as a consequence of PIAS1 knock-down (Figure 4E). Both *MYC *and *LMO2 *have been verified to be responsive to c-Myb knock-down in K562 cells ([49] and unpublished results). Taken together, these observations support our hypothesis that PIAS1 cooperates with c-Myb in a positive fashion to activate the transcription of at least a subset of endogenous c-Myb target genes.

### FLASH, PIAS1 and c-Myb are all co-localized in active RNA polymerase II foci

**Figure 5:**
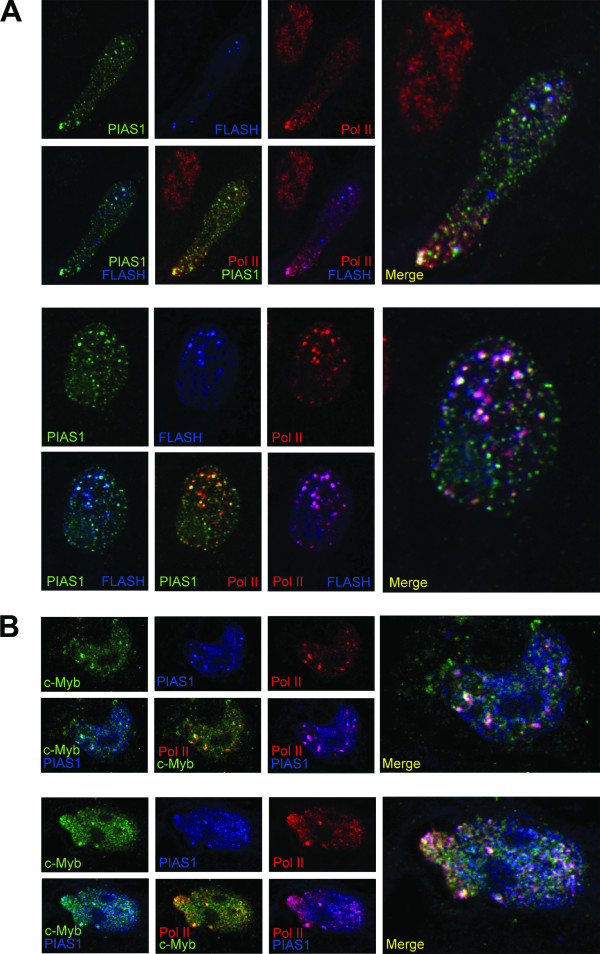
**FLASH, PIAS1 and c-Myb are all co-localized in active RNA polymerase II foci**. **(A) **CV-1 cells were transfected with plasmids encoding HA-PIAS1 and 3×FLAG-FLASH. Cells were analysed by indirect immunofluorescence and confocal microscopy. PIAS1 was detected with rabbit anti-HA antibody and Alexa Fluor 488 goat anti-rabbit IgG (green signal). RNA polymerase II phosphorylated on Ser-5 in the CTD was detected with a mouse monoclonal anti-pol II (8A7) IgM antibody and Alexa Fluor 546 goat anti-mouse IgM, specific for the IgM heavy chains (red signal). FLASH was detected with mouse anti-FLAG antibody and Alexa Fluor 633 goat anti-mouse IgG1, specific for the IgG1 heavy chains (blue signal). Double merged images are shown as indicated, and triple merged images are visualized in right panel (merge). **(B) **CV-1 cells were transfected with plasmids encoding c-Myb-HA and FLAG-PIAS1. c-Myb was detected with rabbit anti-HA antibody and Alexa Fluor 488 goat anti-rabbit IgG (green signal). Active RNA polymerase II was detected as in (A) (red signal). PIAS1 was detected with mouse anti-FLAG antibody and Alexa Fluor 633 goat anti-mouse IgG1, specific for the IgG1 heavy chains (blue signal). Double merged images are shown as indicated, and triple merged images are visualized in right panel (merge).

FLASH is associated with active RNA polymerase II foci, in which we have found FLASH and c-Myb to be co-localized [6]. Since PIAS1 is involved in co-activation of both FLASH and c-Myb, we examined whether PIAS1 also co-localizes with FLASH and c-Myb in these active transcription foci. As shown in Figure 5A, co-transfected FLASH and PIAS1 co-localized with active RNA polymerase II foci. When we analyzed the localization of transfected c-Myb and PIAS1, we observed that although these proteins can be found both in the nucleoplasm and in speckles, they clearly co-localize in some stronger foci. Moreover, these foci co-localize with RNA pol II foci (Figure 5B). In conclusion, FLASH, PIAS1 and c-Myb are all co-localized in active transcription foci.

## Discussion

FLASH and c-Myb are both cancer-related nuclear proteins for which a better understanding of mechanism of action is needed. In this work we have demonstrated a novel link between these two factors through PIAS1. We have earlier reported that FLASH directly interacts with c-Myb and functions as a co-activator of c-Myb [6]. Our search for additional interaction partners of FLASH, led to the identification of PIAS1 as one of the interaction partners of FLASH (Figure 1). Interestingly, PIAS1 enhances the transactivation potential of FLASH through a mechanism that requires the RING domain and therefore presumably the E3 ligase activity of PIAS1 (Figure 2). Moreover, the two proteins both bind to c-Myb and cooperate to enhance its transcriptional activity (Figure 3 and 4). The fact that both FLASH and PIAS1 bind c-Myb suggests the possible formation of a tripartite FLASH-PIAS1-c-Myb complex reinforced by several interaction surfaces, providing a strong enhancing effect on c-Myb-mediated gene activation. Supporting this hypothesis, mutation of the RING domain of PIAS1 or using a truncated protein that do not bind c-Myb, in combination with FLASH, showed a decrease in the enhancement of c-Myb transcriptional activity (Figure 4D). Furthermore, ChIP showed that PIAS1 binds both c-Myb and FLASH supporting a triple complex binding DNA. Finally, we found a close association of FLASH, PIAS1 and c-Myb within active transcription foci (Figure 5), suggesting that FLASH, PIAS1 and c-Myb cooperate to recruit the RNA polymerase II machinery to actively transcribed sites in the genome.

PIAS proteins are well known for their role as inhibitors of STAT proteins and as SUMO E3 ligases (reviewed in [31]). More recently, PIAS proteins have been found to act as transcriptional co-regulators in several systems, a function that may either be activating or repressive, SUMO-dependent or SUMO-independent (reviewed in [31,32]). These functions may also be modulated by specific post-translational modifications such as phosphorylation and methylation [50,51]. Hence, PIAS proteins emerge as sophisticated pleiotrophic transcriptional regulators. In this study we have identified PIAS1 as a novel co-regulator of both FLASH and c-Myb, expanding the range of factors with which PIAS1 physically and functionally interacts. In this regard, our findings parallel the discovery of PIAS1 interacting with the haematopoietic transcription factor GATA-3 where PIAS1 in Th2 cells was found to potentiate GATA-3 mediated activation of cytokine gene promoters [52]. Another interesting parallel is the PIAS3-mediated co-activation of Smad3, where PIAS3 was shown to enhance the transcriptional activity of Smad3 by forming a ternary complex with the co-activator p300 [53]. Like in the present study on PIAS1, PIAS3-mediated co-activation of Smad3 was dependent on an intact RING domain and thus presumably SUMO E3 ligase activity. The detailed mechanism underlying the PIAS1-mediated co-activation of c-Myb is not known, but a role in recruitment is a reasonable assumption. The most obvious hypothesis is that c-Myb binds the promoter of a specific target gene, causing FLASH and PIAS1 to be recruited (Figure 1F and 4B), where PIAS1 functions as a bridge between c-Myb/FLASH and other parts of the transcriptional apparatus, such as p300, general transcription factors or RNA polymerase II. Consistent with this is the observation that PIAS1 interacts with the TATA-binding protein (TBP) [54] and co-localizes with TBP [55] and RNA polymerase II (Figure 5). In this scenario, PIAS1 may act as an assembly factor for transcription complex formation.

It is well established that PIAS proteins act as SUMO E3 ligases (reviewed in [31]). Despite the fact that sumoylation in general is associated with a decrease in the activity of transcription factors, several factors have been reported to be activated by PIAS proteins in a E3-ligase dependent way, as exemplified by Smad3, p53, Rta, IE2 and androgen receptor (AR) [42,43,53,56-59]. FLASH also becomes sumoylated and we have identified the K1813 as major sumoylation site, the modification of which leads to a modest increase in FLASH activity [45]. Based on this, we expected that the PIAS1-mediated increase in FLASH transactivation occurred through FLASH sumoylation. Consistent with this hypothesis, mutation of the RING domain of PIAS1 abolished PIAS1-mediated increase in FLASH activity (Figure 2B). Moreover, when FLASH and PIAS1 were co-expressed, a significant increase in sumoylation of FLASH K1813 was observed (Figure 2A). Still, PIAS1 enhanced the transactivation potential of a FLASH-K1813R mutant, indicating that PIAS1-mediated sumoylation of K1813 is unlikely to be the only mechanism of PIAS1-mediated FLASH activation (Figure 2B). Even though some of the remaining activation may be linked to the existence of other weaker non-identified sumoylation sites in FLASH, or to sumoylation of some unknown partner protein, we cannot exclude the possibility of an alternative mechanism in which PIAS1 co-activation occurs independently of PIAS1-mediated sumoylation. An interesting possibility emerges if the RING finger mutant not only affects the E3-ligase activity of PIAS1, but also its recruitment properties. In the case for the Smad3-PIAS3-p300 interaction, the SUMO E3 ligase activity of PIAS3 was necessary for co-activation, even if the Smad3 SUMO-conjugation sites were not required [53]. More important, the association between PIAS3 and p300 was abolished by a RING finger mutant in PIAS3. If this is a property also of the RING domain in PIAS1, a recruitment mechanism may be more important for PIAS-mediated co-activation than mechanisms dependent on SUMO-conjugation, although both may contribute.

An attractive model of transcription is the transcription factory model, according to which active transcription occurs at discrete sites in the nucleus, termed transcription factories, where multiple active RNA polymerases are concentrated and anchored to a nuclear substructure [60]. Apart from RNAPII, it is not known what components are present in such factories, or what components are required for their formation and function [60]. We have proposed FLASH to be a component of at least a subgroup of transcription factories [6]. The association of FLASH with PIAS1 and our finding that PIAS1 co-localize with FLASH and active RNA polymerase II, suggest that PIAS1 may be an additional component of transcription factories used by c-Myb to orchestrate activation of its target genes.

## Conclusions

In conclusion, this study demonstrates that PIAS1 interacts with FLASH and enhances its co-activation potential. Both FLASH and PIAS1 associate with c-Myb and cooperate in enhancing c-Myb-dependent gene activation. FLASH, PIAS1 and c-Myb are all closely associated with active RNA polymerase II in nuclear foci resembling transcription factories. Hence, our study strengthens the link between two cancer-related nuclear factors, c-Myb and FLASH, through their common interaction with PIAS1.

## Methods

### Yeast two-hybrid screening and interaction assays

The Y2H screening with pDBT-FLASH-D as bait was performed as described [45]. Positive clones were validated in the Y2H assay by retransformation and checking for activation of the *HIS3*, *ADE2 *and *LacZ *reporter genes. The identities of isolated clones were determined by DNA sequencing. For verification of the interaction, bait and prey plasmids were retransformed in AH109 and Y187 respectively, subjected to mating and subsequent reporter activation testing.

### Plasmids

pDBT-FLASH-D was used as bait in the Y2H screening. It encodes amino acids 1508-1982 of human FLASH fused to Gal4p-DBD. pDBT-FLASH-A, -B, -C, D and -E encode different FLASH fragments in fusion with Gal4p-DBD (A; amino acids 1-304, B: 305-867, C: 868-1507, D: 1508-1982 and E: 1814-1982). pDBT-c-Myb encodes full-length human c-Myb in fusion with Gal4p-DBD [25]. pGADT7-PIAS1[1-501] encodes amino acids 1-501 of human PIAS1 in fusion with the transactivation domain of Gal4p, and was isolated in the two-hybrid screening. pGADT7-PIAS1 encodes full-length human PIAS1 in fusion with Gal4p-AD. pGEX-6p-2-FLASH-A is encoding GST fused to the FLASH A fragment (amino acid residues 1-304). pGEX-6p-2-FLASH-D is encoding GST fused to the FLASH D fragment (amino acid residues 1508-1982), while pGEX-6p-2-FLASH-D-KR has the SUMO acceptor lysine K1813 mutated to arginine. pHA-FLASH encoding full-length mouse FLASH with HA-tag was kindly provided by Y.K. Jung [61]. pCIneo-3×FLAG-FLASH encodes full-length human FLASH with an N-terminal triple FLAG-tag. pCIneo-3×FLAG-FLASH-D encodes amino acids 1508-1982 of FLASH with an N-terminal triple FLAG-tag, while pCIneo-3×FLAG-FLASH-D-K1813R encodes the same part of FLASH with a K1813R mutation [45]. pGFP-FLASH encodes a GFP-FLASH fusion protein and was a kind gift from V. De Laurenzi [5]. pCIneo-hcM encodes human c-Myb [25]. pCIneo-hcM-HA-2KR encodes human c-Myb with a C-terminal HA-tag and with sumoylation sites K503 and K527 mutated to arginine [25]. The expression vector pCIneoB-GBD2-hcM[233-640]-HA, encoding a c-Myb protein lacking its own DBD in fusion Gal4p-DBD, has been described [26]. pCIneo-H6-hSUMO1 encodes human SUMO-1 with a N-terminal histidine tag. pGFP-SUMO-1 encodes a GFP-SUMO-1 fusion protein and was kindly provided by G. Del Sal [62]. pCIneoB-3×FLAG-PIAS1 and pCIneoB-3×FLAG-PIAS1 RING finger mutant encode human PIAS1 wild-type and PIAS1 with RING finger mutations (C346S/C351S/H353A/C356S), respectively, both with an N-terminal triple FLAG-tag. pCMV5-FLAG-PIAS1 and pCMV5-FLAG-PIAS1[C350S] encode PIAS1 wild-type and a RING finger mutant, respectively. Both have an N-terminal FLAG-tag and were kind gifts from V. De Laurenzi [44]. pcDNA3-HA-hPIAS1 encodes PIAS1 with an N-terminal HA-tag. The Myb-responsive reporter plasmid pGL4b-3×MRE(GG)-MYC-aab contains three Myb-responsive elements and core promoter from *MYC *(P2 promoter) upstream the luciferase reporter gene [26]. The Gal4p-responsive reporter plasmid pGL3b-5×GRE-SNRPN is described in [29]. pCIneo-GBD1-FLASH and pCIneo-GBD1-FLASH-KR encode Gal4p-DNA-binding domain in fusion with full-length wild-type FLASH [6] and FLASH-K1813R [45] respectively. All constructs generated by PCR were verified by sequencing. Primer sequences are available upon request.

### GST pulldown assays

GST, GST-FLASH-A, GST-FLASH-D and GST-FLASH-D-KR were expressed in *E. coli *[29,63]. GST pulldown was performed as described earlier in cell extracts from transfected COS-1 cells [6]. The bound proteins were eluted by boiling in SDS sample buffer, subjected to SDS-PAGE, and detected by immunoblotting as described earlier [6].

### Cell culture and transient transfections

CV-1 and COS-1 cells were grown in DMEM (Invitrogen) supplemented with antibiotics, L-glutamine and 10% foetal bovine serum (FBS). HD11 cells were grown in IMDM supplemented with antibiotics and 10% serum (8% FBS and 2% chicken serum). K562 cells were cultivated in IMDM supplemented with 2 mM glutamax, antibiotics and 10% FBS. All four cell lines were kept at 37°C in a humidified atmosphere of 5% CO_2 _in air. Transient transfections were performed using FuGENE6 Transfection Reagent (Roche).

### Immunoprecipitation

Transfected COS-1 cells (15-cm dishes; 2.5 × 10^6 ^cells/dish; 1 dish per IP) were harvested 24 h after transfection in 150 μl of lysis buffer (400 mM NaCl, 1.0% Triton X-100, 10% glycerol, 50 mM Tris pH 8.0, 1 mM EDTA, 100 mM NaF, 1 mM MgCl_2_, 10 mM DTT, and Complete Protease Inhibitor), debris was removed by centrifugation and the cleared lysate was diluted 1:4 in dilution buffer (lysis buffer without NaCl and Triton X-100). Then 600 μl of diluted lysate (100 mM NaCl, 0.25% Triton X-100, 10% glycerol, 50 mM Tris pH 8.0, 1 mM EDTA, 100 mM NaF, 1 mM MgCl_2_) was subjected to immunoprecipitation with indicated antibodies and protein G-Sepharose beads after a preclearing step with G-Sepharose beads only. Immunoprecipitation was performed on a roller at 4°C overnight. The beads were washed three times in 500 μl of wash buffer (100 mM NaCl, 0.25% Triton X-100, 10% glycerol, 50 mM Tris pH 8.0, 1 mM EDTA, 100 mM NaF, 1 mM MgCl_2_), and the proteins eluted in 40 μl SDS loading buffer for 4 min at 95°C. Proteins were separated by SDS-PAGE and detected with immunoblotting.

### Chromatin immunoprecipitation

Transfected HEK-293 cells, C#1 [41], were cross-linked with 1% formaldehyde in PBS at room temperature for 15 min. Cross-linking was performed with rotation, and the reaction was stopped by addition of glycine to a final concentration of 125 mM. After two washes with PBS, cells were lysed in IP buffer (50 mM Tris-HCl pH 7.5, 5 mM EDTA, 1% Triton, 0.5% NP-40, 150 mM NaCl, 1.0% SDS and Complete Protease Inhibitor) and frozen in LN_2_. After thawing the samples were diluted to a final SDS concentration of 0.1%. Samples were sonicated to generate sheared DNA fragments around 400 base pairs (soluble chromatin fraction), and insoluble chromatin was discarded after centrifugation. Dynabeads™ ProteinG were washed with PBS and incubated with antibody at room temperature for 40 min followed by washing with PBS. The soluble chromatin fraction was then added followed by incubation overnight at 4°C with rotation. Chromatin equivalent to 200 000 cells was used per IP with 20 μl Dynabeads™ ProteinG and 2 μg antibody, in a total volume of 1.2 ml IP buffer. The immunoprecipitates were washed five times in IP buffer, before DNA was eluted with 1% SDS in 100 mM sodium carbonate at 65°C for 10 min. After treatment with RNAse A and proteinase K, cross-linking was reversed by incubation at 65°C for 8 h. DNA was purified using silica columns (Macherey-Nagel) and eluted in 50 µl 10 mM Tris-HCl [pH 7.5]. 2.5 µl of the eluted DNA was used as template for quantitative real-time PCR in a total volume of 20 µl (LightCycler® 480 SYBR Green I Master, Roche Diagnostics). Standard curves of genomic DNA were run alongside the ChIP samples for each primer pair, and analyzed on a LightCycler® 480 (Roche Diagnostics). Input DNA was used to normalize values from ChIP samples.

### Antibodies

For Western immunoblotting the following antibodies were used: rabbit anti-HA (H 6908, Sigma-Aldrich), mouse anti-FLAG M2 antibody (F 3165, Sigma-Aldrich), goat anti-PIAS1 (sc-8152, Santa Cruz), rabbit anti-PIAS1 (ab77231, Abcam) rabbit anti-GFP (ab6556, Abcam), mouse anti-GAPDH (Biodesign International, Saco, Maine), and mouse anti-tubulin (T9026, Sigma). Anti-mouse IgG-HRP (NA 931, GE Healthcare Life Sciences), anti-rabbit IgG-HRP (NA 934, GE Healthcare Life Sciences), and anti-goat IgG-HRP (sc-2033, Santa Cruz) were used as secondary antibodies. As immunofluorescence antibodies rabbit anti-HA (H 6908, Sigma-Aldrich), mouse anti-FLAG M2 antibody (F 3165, Sigma-Aldrich), and mouse anti-pol II (8A7, sc-13583, Santa Cruz) were used. Alexa Fluor 488 goat anti-rabbit IgG(H+L), Alexa 546 goat anti-mouse IgM(μ), and Alexa Fluor 633 goat anti-mouse IgG1 (γ1) (Molecular Probes) were used as secondary antibodies.

### Reporter gene assays

CV-1 cells were plated in 24-well microplates at a concentration of 2×10^4 ^cells per well the day before transfection. The cells were transfected with a total of 0.8 μg DNA per well. Cells were washed twice in PBS, and lysed in Passive Lysis Buffer (Promega) 24 hours after transfection. Luciferase activity was monitored with a Luciferase assay kit (Promega). Light emission was determined with a luminometer (Turner Designs). Each experiment was performed in triplicate, and average data from three independent transfection experiments are presented.

### RNA isolation and quantitative RT-PCR

HD11 cells were transfected as described above with a total of 5 µg DNA per well in 6-well microplates seeded with 5×10^5 ^cells per well the day before. 24 hours after transfection the cells were harvested and total RNA was extracted with Trizol reagent (Invitrogen), followed by DNase treatment and purification of the RNA using RNeasy columns (Qiagen). 3µg of RNA for each sample were used for reverse transcription using the Superscript™III system (Invitrogen). Two different dilutions of the cDNA obtained were subjected to real-time PCR analysis to determine the expression of the c-Myb target gene *mim*-1, using the LightCycler DNA MasterPlus SYBR Green Kit (Roche). A standard curve made from serial dilutions of cDNA was used to calculate the relative amount of *mim-1 *mRNAs in each sample. These values were normalized to the relative amount of the reference gene *HPRT *in the same samples, calculated from a standard curve established in the same way. The cellular transfections were performed in triplicate and the experiment was repeated three times. Primer sequences are available upon request.

### RNA interference

RNA interference was performed as previously described [29]. The K562 cells were transfected with FlexiTube siRNA from Qiagen Hs_PIAS1_1 or Ctrl_LuciferaseGL2_2 at 5 pmol/sample. After 24 hour RNA were isolated and analysed in quantitative RT-PCR, essentially as described above. Target genes evaluated were *PIAS1*, *LMO2 *and *MYC*, and as reference genes *ACTB *and *POLR2A*. The primer sequences are available upon request.

### Immunofluorescence and confocal laser scanning microscopy

1.8×10^4 ^CV-1 cells were plated out in 24-well microplates containing cover-slips and transfected with a total of 0.6 μg DNA. 24 hours after transfection cells were washed in PBS. Cells were fixed and permeabilized with ice cold methanol for 5 min. Samples were washed three times for 5 min in PBS containing 0.1% Tween 20, then blocked for 30 min with 2% BSA in PBS with 0.1% Tween 20, following incubation with primary antibodies diluted 1:50 in the blocking solution for 45 min. Samples were then washed three times as above, and incubated with secondary antibodies diluted 1:100 in the blocking solution for 30 min. Samples were washed three times again and incubated with Hoechst 33258 (Sigma-Aldrich) for 20 min to visualize DNA. Samples were washed once in PBS containing 0.1% Tween 20, once in PBS and once in dH_2_O. The cover-slips were then placed on microscope slides using mounting medium (Dako). Cells were examined using a FluoView laser scanning system from Olympus. Images from the different channels were collected sequentially to prevent bleed through.

## Competing interests

The authors declare that they have no competing interests.

## Authors' contributions

AHAK designed, carried out the majority of experiments, interpreted and analyzed the results, and wrote the manuscript. PIL performed a subset of the experiments and contributed to the writing of the manuscript. AKM performed the sumoylation assays and ChIP, and VM did the RNAi experiments. ML performed pulldowns and ChIP, and TS performed the co-immunoprecipitations and revised the manuscript, while OSG designed the project and supervised the experiments. All authors read and approved the final manuscript.

## References

[B1] Remke M Pfister S Kox C Toedt G Becker N Benner A Werft W Breit S Liu S Engel F High-resolution genomic profiling of childhood T-ALL reveals frequent copy-number alterations affecting the TGF-beta and PI3K-AKT pathways and deletions at 6q15-16.1 as a genomic marker for unfavorable early treatment response Blood 2009 114 1053 1062 10.1182/blood-2008-10-186536 19406988

[B2] Park TS Lee SG Song J Lee KA Kim J Choi JR Lee ST Marschalek R Meyer C CASP8AP2 is a novel partner gene of MLL rearrangement with t(6;11)(q15;q23) in acute myeloid leukemia Cancer Genet Cytogenet 2009 195 94 95 10.1016/j.cancergencyto.2009.06.023 19837277

[B3] Imai Y Kimura T Murakami A Yajima N Sakamaki K Yonehara S The CED-4-homologous protein FLASH is involved in Fas-mediated activation of caspase-8 during apoptosis Nature 1999 398 777 785 10.1038/19709 10235259

[B4] Milovic-Holm K Krieghoff E Jensen K Will H Hofmann TG FLASH links the CD95 signaling pathway to the cell nucleus and nuclear bodies EMBO J 2007 26 391 401 10.1038/sj.emboj.7601504 17245429 PMC1783462

[B5] Barcaroli D Dinsdale D Neale MH Bongiorno-Borbone L Ranalli M Munarriz E Sayan AE McWilliam JM Smith TM Fava E FLASH is an essential component of Cajal bodies Proc Natl Acad Sci USA 2006 103 14802 14807 10.1073/pnas.0604225103 17003126 PMC1578500

[B6] Alm-Kristiansen AH Saether T Matre V Gilfillan S Dahle O Gabrielsen OS FLASH acts as a co-activator of the transcription factor c-Myb and localizes to active RNA polymerase II foci Oncogene 2008 27 4644 4656 10.1038/onc.2008.105 18408764

[B7] Barcaroli D Bongiorno-Borbone L Terrinoni A Hofmann TG Rossi M Knight RA Matera AG Melino G De Laurenzi V FLASH is required for histone transcription and S-phase progression Proc Natl Acad Sci USA 2006 103 14808 14812 10.1073/pnas.0604227103 17003125 PMC1578501

[B8] Bongiorno-Borbone L De Cola A Vernole P Finos L Barcaroli D Knight RA Melino G De Laurenzi V FLASH and NPAT positive but not Coilin positive Cajal Bodies correlate with cell ploidy Cell Cycle 2008 7 2357 2367 18677100 10.4161/cc.6344

[B9] Yang XC Burch BD Yan Y Marzluff WF Dominski Z FLASH, a proapoptotic protein involved in activation of caspase-8, is essential for 3' end processing of histone pre-mRNAs Mol Cell 2009 36 267 278 10.1016/j.molcel.2009.08.016 19854135 PMC2819824

[B10] Bongiorno-Borbone L De Cola A Barcaroli D Knight RA Di Ilio C Melino G De Laurenzi V FLASH degradation in response to UV-C results in histone locus bodies disruption and cell-cycle arrest Oncogene 2010 29 802 810 10.1038/onc.2009.388 19915611

[B11] Kino T Chrousos GP Tumor necrosis factor alpha receptor- and Fas-associated FLASH inhibit transcriptional activity of the glucocorticoid receptor by binding to and interfering with its interaction with p160 type nuclear receptor coactivators J Biol Chem 2003 278 3023 3029 10.1074/jbc.M209234200 12477726

[B12] Kino T Ichijo T Chrousos GP FLASH interacts with p160 coactivator subtypes and differentially suppresses transcriptional activity of steroid hormone receptors J Steroid Biochem Mol Biol 2004 92 357 363 10.1016/j.jsbmb.2004.09.003 15698540

[B13] Obradovic D Tirard M Nemethy Z Hirsch O Gronemeyer H Almeida OF DAXX, FLASH, and FAF-1 modulate mineralocorticoid and glucocorticoid receptor-mediated transcription in hippocampal cells--toward a basis for the opposite actions elicited by two nuclear receptors? Mol Pharmacol 2004 65 761 769 10.1124/mol.65.3.761 14978255

[B14] Oh IH Reddy EP The myb gene family in cell growth, differentiation and apoptosis Oncogene 1999 18 3017 3033 10.1038/sj.onc.1202839 10378697

[B15] Ramsay RG Gonda TJ MYB function in normal and cancer cells Nat Rev Cancer 2008 8 523 534 10.1038/nrc2439 18574464

[B16] Lahortiga I De Keersmaecker K Van Vlierberghe P Graux C Cauwelier B Lambert F Mentens N Beverloo HB Pieters R Speleman F Duplication of the MYB oncogene in T cell acute lymphoblastic leukemia Nat Genet 2007 39 593 595 10.1038/ng2025 17435759

[B17] Clappier E Cuccuini W Kalota A Crinquette A Cayuela JM Dik WA Langerak AW Montpellier B Nadel B Walrafen P The C-MYB locus is involved in chromosomal translocation and genomic duplications in human T-cell acute leukemia (T-ALL), the translocation defining a new T-ALL subtype in very young children Blood 2007 110 1251 1261 10.1182/blood-2006-12-064683 17452517

[B18] Persson M Andren Y Mark J Horlings HM Persson F Stenman G Recurrent fusion of MYB and NFIB transcription factor genes in carcinomas of the breast and head and neck Proc Natl Acad Sci USA 2009 106 18740 18744 10.1073/pnas.0909114106 19841262 PMC2773970

[B19] Zhao H Kalota A Jin S Gewirtz AM The c-myb proto-oncogene and microRNA-15a comprise an active autoregulatory feedback loop in human hematopoietic cells Blood 2009 113 505 516 10.1182/blood-2008-01-136218 18818396 PMC2628359

[B20] Andersson KB Kowenz-Leutz E Brendeford EM Tygsett AH Leutz A Gabrielsen OS Phosphorylation-dependent Down-regulation of c-Myb DNA Binding Is Abrogated by a Point Mutation in the v-myb Oncogene J Biol Chem 2003 278 3816 3824 10.1074/jbc.M209404200 12456674

[B21] Ganter B Lipsick JS Myb and oncogenesis Adv Cancer Res 1999 76 21 60 full_text 10218098 10.1016/s0065-230x(08)60773-3

[B22] Winn LM Lei W Ness SA Pim-1 phosphorylates the DNA binding domain of c-Myb Cell Cycle 2003 2 258 262 10.4161/cc.2.3.383 12734436

[B23] Matre V Nordgard O Alm-Kristiansen AH Ledsaak M Gabrielsen OS HIPK1 interacts with c-Myb and modulates its activity through phosphorylation Biochem Biophys Res Commun 2009 388 150 154 10.1016/j.bbrc.2009.07.139 19646965

[B24] Bies J Markus J Wolff L Covalent attachment of the SUMO-1 protein to the negative regulatory domain of the c-Myb transcription factor modifies its stability and transactivation capacity J Biol Chem 2002 277 8999 9009 10.1074/jbc.M110453200 11779867

[B25] Dahle O Andersen TO Nordgard O Matre V Del Sal G Gabrielsen OS Transactivation properties of c-Myb are critically dependent on two SUMO-1 acceptor sites that are conjugated in a PIASy enhanced manner Eur J Biochem 2003 270 1338 1348 10.1046/j.1432-1033.2003.03504.x 12631292

[B26] Molvaersmyr AK Saether T Gilfillan S Lorenzo PI Kvaloy H Matre V Gabrielsen OS A SUMO-regulated activation function controls synergy of c-Myb through a repressor-activator switch leading to differential p300 recruitment Nucleic Acids Res 2010 38 4970 4984 10.1093/nar/gkq245 20385574 PMC2926607

[B27] Sano Y Ishii S Increased affinity of c-Myb for CREB-binding protein (CBP) after CBP-induced acetylation J Biol Chem 2001 276 3674 3682 10.1074/jbc.M006896200 11073948

[B28] Tomita A Towatari M Tsuzuki S Hayakawa F Kosugi H Tamai K Miyazaki T Kinoshita T Saito H c-Myb acetylation at the carboxyl-terminal conserved domain by transcriptional co-activator p300 Oncogene 2000 19 444 451 10.1038/sj.onc.1203329 10656693

[B29] Saether T Berge T Ledsaak M Matre V Alm-Kristiansen AH Dahle O Aubry F Gabrielsen OS The chromatin remodeling factor Mi-2alpha acts as a novel co-activator for human c-Myb J Biol Chem 2007 282 13994 14005 10.1074/jbc.M700755200 17344210

[B30] Saether T Pattabiraman DR Alm-Kristiansen AH Vogt-Kielland LT Gonda TJ Gabrielsen OS A functional SUMO-interacting motif in the transactivation domain of c-Myb regulates its myeloid transforming ability Oncogene 2011 30 212 222 10.1038/onc.2010.397 20802522

[B31] Schmidt D Muller S PIAS/SUMO: new partners in transcriptional regulation Cell Mol Life Sci 2003 60 2561 2574 10.1007/s00018-003-3129-1 14685683 PMC11138616

[B32] Sharrocks AD PIAS proteins and transcriptional regulation--more than just SUMO E3 ligases? Genes Dev 2006 20 754 758 10.1101/gad.1421006 16600908

[B33] Huang CY Beliakoff J Li X Lee J Sharma M Lim B Sun Z hZimp7, a novel PIAS-like protein, enhances androgen receptor-mediated transcription and interacts with SWI/SNF-like BAF complexes Mol Endocrinol 2005 19 2915 2929 10.1210/me.2005-0097 16051670

[B34] Sharma M Li X Wang Y Zarnegar M Huang CY Palvimo JJ Lim B Sun Z hZimp10 is an androgen receptor co-activator and forms a complex with SUMO-1 at replication foci EMBO J 2003 22 6101 6114 10.1093/emboj/cdg585 14609956 PMC275443

[B35] Shuai K Liu B Regulation of gene-activation pathways by PIAS proteins in the immune system Nat Rev Immunol 2005 5 593 605 10.1038/nri1667 16056253

[B36] Gill G Something about SUMO inhibits transcription Curr Opin Genet Dev 2005 15 536 541 10.1016/j.gde.2005.07.004 16095902

[B37] Hay RT SUMO: a history of modification Mol Cell 2005 18 1 12 10.1016/j.molcel.2005.03.012 15808504

[B38] Lee H Quinn JC Prasanth KV Swiss VA Economides KD Camacho MM Spector DL Abate-Shen C PIAS1 confers DNA-binding specificity on the Msx1 homeoprotein Genes Dev 2006 20 784 794 10.1101/gad.1392006 16600910 PMC1472282

[B39] Matsuura T Shimono Y Kawai K Murakami H Urano T Niwa Y Goto H Takahashi M PIAS proteins are involved in the SUMO-1 modification, intracellular translocation and transcriptional repressive activity of RET finger protein Exp Cell Res 2005 308 65 77 10.1016/j.yexcr.2005.04.022 15907835

[B40] Sachdev S Bruhn L Sieber H Pichler A Melchior F Grosschedl R PIASy, a nuclear matrix-associated SUMO E3 ligase, represses LEF1 activity by sequestration into nuclear bodies Genes Dev 2001 15 3088 3103 10.1101/gad.944801 11731474 PMC312834

[B41] Stielow B Sapetschnig A Wink C Kruger I Suske G SUMO-modified Sp3 represses transcription by provoking local heterochromatic gene silencing EMBO Rep 2008 9 899 906 10.1038/embor.2008.127 18617891 PMC2529347

[B42] Kotaja N Karvonen U Janne OA Palvimo JJ PIAS proteins modulate transcription factors by functioning as SUMO-1 ligases Mol Cell Biol 2002 22 5222 5234 10.1128/MCB.22.14.5222-5234.2002 12077349 PMC139781

[B43] Lee JM Kang HJ Lee HR Choi CY Jang WJ Ahn JH PIAS1 enhances SUMO-1 modification and the transactivation activity of the major immediate-early IE2 protein of human cytomegalovirus FEBS Lett 2003 555 322 328 10.1016/S0014-5793(03)01268-7 14644436

[B44] Munarriz E Barcaroli D Stephanou A Townsend PA Maisse C Terrinoni A Neale MH Martin SJ Latchman DS Knight RA PIAS-1 is a checkpoint regulator which affects exit from G1 and G2 by sumoylation of p73 Mol Cell Biol 2004 24 10593 10610 10.1128/MCB.24.24.10593-10610.2004 15572666 PMC533962

[B45] Alm-Kristiansen AH Norman IL Matre V Gabrielsen OS SUMO modification regulates the transcriptional activity of FLASH Biochem Biophys Res Commun 2009 387 494 499 10.1016/j.bbrc.2009.07.053 19615980

[B46] Sramko M Markus J Kabat J Wolff L Bies J Stress-induced inactivation of the c-Myb transcription factor through conjugation of SUMO-2/3 proteins J Biol Chem 2006 281 40065 40075 10.1074/jbc.M609404200 17077080

[B47] Schmidt M Nazarov V Stevens L Watson R Wolff L Regulation of the resident chromosomal copy of c-myc by c-Myb is involved in myeloid leukemogenesis Mol Cell Biol 2000 20 1970 1981 10.1128/MCB.20.6.1970-1981.2000 10688644 PMC110814

[B48] Bianchi E Zini R Salati S Tenedini E Norfo R Tagliafico E Manfredini R Ferrari S c-myb supports erythropoiesis through the transactivation of KLF1 and LMO2 expression Blood 2010 116 e99 110 10.1182/blood-2009-08-238311 20686118

[B49] Berge T Matre V Brendeford EM Saether T Luscher B Gabrielsen OS Revisiting a selection of target genes for the hematopoietic transcription factor c-Myb using chromatin immunoprecipitation and c-Myb knockdown Blood Cells Mol Dis 2007 39 278 286 10.1016/j.bcmd.2007.05.007 17587615

[B50] Liu B Yang Y Chernishof V Loo RR Jang H Tahk S Yang R Mink S Shultz D Bellone CJ Proinflammatory stimuli induce IKKalpha-mediated phosphorylation of PIAS1 to restrict inflammation and immunity Cell 2007 129 903 914 10.1016/j.cell.2007.03.056 17540171

[B51] Weber S Maass F Schuemann M Krause E Suske G Bauer UM PRMT1-mediated arginine methylation of PIAS1 regulates STAT1 signaling Genes Dev 2009 23 118 132 10.1101/gad.489409 19136629 PMC2632166

[B52] Zhao X Zheng B Huang Y Yang D Katzman S Chang C Fowell D Zeng WP Interaction between GATA-3 and the transcriptional coregulator Pias1 is important for the regulation of Th2 immune responses J Immunol 2007 179 8297 8304 18056374 10.4049/jimmunol.179.12.8297

[B53] Long J Wang G Matsuura I He D Liu F Activation of Smad transcriptional activity by protein inhibitor of activated STAT3 (PIAS3) Proc Natl Acad Sci USA 2004 101 99 104 10.1073/pnas.0307598100 14691252 PMC314145

[B54] Prigge JR Schmidt EE Interaction of protein inhibitor of activated STAT (PIAS) proteins with the TATA-binding protein, TBP J Biol Chem 2006 281 12260 12269 10.1074/jbc.M510835200 16522640 PMC2030495

[B55] Du JX Yun CC Bialkowska A Yang VW Protein inhibitor of activated STAT1 interacts with and up-regulates activities of the pro-proliferative transcription factor Kruppel-like factor 5 J Biol Chem 2007 282 4782 4793 10.1074/jbc.M603413200 17178721 PMC2212600

[B56] Chang LK Lee YH Cheng TS Hong YR Lu PJ Wang JJ Wang WH Kuo CW Li SS Liu ST Post-translational modification of Rta of Epstein-Barr virus by SUMO-1 J Biol Chem 2004 279 38803 38812 10.1074/jbc.M405470200 15229220

[B57] Megidish T Xu JH Xu CW Activation of p53 by protein inhibitor of activated Stat1 (PIAS1) J Biol Chem 2002 277 8255 8259 10.1074/jbc.C200001200 11788578

[B58] Yang SH Sharrocks AD PIASx acts as an Elk-1 coactivator by facilitating derepression EMBO J 2005 24 2161 2171 10.1038/sj.emboj.7600690 15920481 PMC1150884

[B59] Lee J Beliakoff J Sun Z The novel PIAS-like protein hZimp10 is a transcriptional co-activator of the p53 tumor suppressor Nucleic Acids Res 2007 35 4523 4534 10.1093/nar/gkm476 17584785 PMC1935018

[B60] Sutherland H Bickmore WA Transcription factories: gene expression in unions? Nat Rev Genet 2009 10 457 466 10.1038/nrg2592 19506577

[B61] Choi YH Kim KB Kim HH Hong GS Kwon YK Chung CW Park YM Shen ZJ Kim BJ Lee SY Jung YK FLASH coordinates NF-kappa B activity via TRAF2 J Biol Chem 2001 276 25073 25077 10.1074/jbc.M102941200 11340079

[B62] Gostissa M Hengstermann A Fogal V Sandy P Schwarz SE Scheffner M Del Sal G Activation of p53 by conjugation to the ubiquitin-like protein SUMO-1 EMBO J 1999 18 6462 6471 10.1093/emboj/18.22.6462 10562558 PMC1171709

[B63] Gabrielsen OS Sentenac A Fromageot P Specific DNA binding by c-Myb: evidence for a double helix-turn-helix- related motif Science 1991 253 1140 1143 10.1126/science.1887237 1887237

